# Testosterone deficiency and metabolic disturbances in men who fathered a child by use of donated spermatozoa

**DOI:** 10.1038/s41598-022-17864-y

**Published:** 2022-08-24

**Authors:** Angel Elenkov, Peter Zarén, Bianca Sundell, Lovisa Lundin, Aleksander Giwercman

**Affiliations:** 1 Reproductive Medicine Centre, Skane University Hospital Malmo, Malmö, Sweden; 2 Department of Translational Medicine, Clinical Research Centre, Lund University, Jan Waldenströms gata 35, Building 60, Plan 9, 20502 Malmö, Sweden

**Keywords:** Gonadal disorders, Hypogonadism, Endocrine reproductive disorders

## Abstract

Dose–response association between level of impairment of semen quality and risk of morbidity or premature death has been reported. Therefore, it can be presumed that men utilizing donated spermatozoa, i.e. patients with non-obstructive azoospermia, are at highest risk for adverse health outcomes. To evaluate the risks of prescription of medications for common metabolic disturbances and testosterone replacement therapy (TRT) among men who father children with donated spermatozoa—who presumably do it due to severe impairment of fertility. We used Swedish nationwide register data on all fathers who had a live-born child between 2007 and 2014 in order to compare men who fathered children with donated spermatozoa to the ones who became fathers by using own gametes. Cox regression analysis was used in order to estimate the post-conception incidence of prescription of medicines for hypertension (HT), diabetes (type 1 and 2), dyslipidaemia (DLE) or TRT. Starting the follow up at time of conception, models were adjusted for age, educational level, and previous cancer treatment. In total 410,119 childbirths were included in the analysis. Among them, for 390 fathers donated spermatozoa were utilized. Fathers to children conceived with donated spermatozoa had higher risk for having TRT prescribed (HR: 18.14; 95%CI: 11.71–28.10; *p* ≪ 0.001). Same was true for DLE (HR: 2.08; 95%CI: 1.27–3.39; *p* = 0.003) but not diabetes. Fathers to children conceived by use of donated spermatozoa are at significantly increased risk for testosterone treatment and dyslipidaemia, necessitating stringent follow up and inclusion in prevention programs.

## Introduction

Infertility affects 15% of all couples and impairment of male reproductive function is a contributing factor in at least 50% of the cases. In recent years, male infertility has been repeatedly reported to be associated with higher morbidity and mortality due to non-communicable diseases^1–3^. Impaired sperm quality and reduced fertility have, in a dose–response manner, been associated with higher rates of premature death, suggesting that the degree of spermatogenic failure is positively associated with risk of adverse health outcomes^4,5^. This model would suggest that men with the most severe cases of male infertility i.e., non-obstructive azoospermia (NOA)—total lack of sperm in the ejaculate or extremely low sperm counts—can be expected to be at highest health risk.

Despite the fact that these men represent approximately 1% of the male population and 10–15% of the cases of male infertility^6^, studies focusing on their long-term morbidity are very scarce and the data are inconclusive. In order to address such a question, ideally, a big cohort with available semen analysis data is needed. In practice such data sets are very scarce, small, incomplete or have short follow up. However, in Sweden, access to national register data provide a unique option for studying long-term health outcomes in this subgroup of men. We hypothesised that men who utilise donated sperm for parenthood do it due to NOA or severe oligozoospermia. Therefore, we have use of donated spermatozoa for fertility treatment as a proxy for identifying men with NOA or severe olizoospermia in Swedish population registries.

In many clinics dealing with assisted reproduction, endocrinological and metabolic assessment is not a part of routine investigation of males in couples using donated sperms for infertility treatment. In order to evaluate whether those men should be a target of such investigations, we have used Swedish national registry data. We evaluated the prospective risks of prescription of medications for common cardio-metabolic disturbances such as hypertension, diabetes and dyslipidaemia among fathers who used donated spermatozoa for child conception. Previous research has shown the infertile men are more likely to be hypogonadal^7,8^. Low testosterone is known to be associated with cardiometabolic disturbances and higher mortality and can possibly serve as a mediator for the associations between infertility and adult-onset diseases^9–12^. Therefore, we also calculated the risk of *new* prescription of testosterone replacement therapy (TRT) among men who used donated spermatozoa in order to father a child. We compared these men to those who became fathers without use of donated spermatozoa.

## Materials and methods

We used nationwide register data in order to identify the fathers of all life-born children in Sweden between 2007 and 2014. By using the unique identity number given to each Swedish citizen we combined several registers: Medical Birth Register, Multi Generation Register, Prescription Drug register (SPDR) and Swedish Register for Assisted Reproduction (Q-IVF). Since the start of the Q-IVF register in 2007, all treatments by public or private providers in Sweden utilising reproductive technologies are reported to Q-IVF including the origin of used gametes^13^. Prescription medicines are issued in Sweden using specially developed online platform and from 2005 onwards all drugs dispensed via the pharmacies are reported to SPDR^14^.

In Sweden all inhabitants have a unique 10-digit personal number. In order to preserve anonymity, all cross-linking between the registries were done by the Swedish National Board of Health and Welfare and, subsequently, before the data were given to the researchers, the 10-digit personal number was replaced by a code not allowing identification of study subjects.

The study has been approved by the Regional Ethical Board in Lund (No: 2015/670). all methods were performed in accordance with the relevant guidelines and regulations.

We identified four groups of prescribed medicines to all fathers—medicines for hypertension and cardiovascular disease (AHT), diabetes mellitus (DM, type 1 + 2), drugs for dyslipidemia (DLE) and TRT as previously described^15,16^. According to Adult Treatment Panel III (ATPIII) men who have at least three of the following criteria: waist circumference over 40 inches, blood pressure over 130/85 mmHg, fasting triglyceride level over 150 mg/dl, fasting high-density lipoprotein (HDL) cholesterol level less than 40 mg/dl and fasting blood sugar over 100 mg/dl are diagnosed with metabolic syndrome (MetS). We used three filled prescriptions (one for DM, AHT and DLE) for one man to serve as proxy for MetS.

From MBR and Q-IVF data on birth date and gestational age were available, allowing us to estimate the approximate date of child conception.

### Statistical analysis

Baseline characteristics were analysed using descriptive statistics presenting means and standard deviations (SD). As exposure group we used all men who have had children with donated spermatozoa. Firstly, we compared them to all men who have fathered children spontaneously. Previously we reported higher risk of MetS prescriptions among men who father children with help of ICSI^15^. Therefore, a sub-analysis, with men who fathered children using ICSI with own gametes as controls, was performed.

Hazard ratios (HR) and 95% confidence intervals (CI) for drug prescription were calculated using the Cox proportional hazards model with accompanying Kaplan–Meier curves. In order to exclude bias by multiple inclusion of the same individuals, we included only the first-born child during the study period in each analysis. Power analysis for was performed using ANOVA. The results showed sufficient sample size (1 − β > 0.99 for all outcomes in the primary analysis and for TRT in the sub analysis. In the latter estimates showed 1 − β = 0.95 for MetS, 1 − β = 0.55 for AHT, 1 − β = 0.96 for DLE, and 1 − β = 0.65 for AD).

Time on study was defined as timescale with men entering at time of conception of their child adjusting for age, educational level, (4 categories − 10 years or less; 10–14; 15 or more years or missing data). Information on history of cancer (any malignancy: yes/no) before child conception was also included to the models since these men have been shown to have higher rates of hypogonadism and gonadal dysfunction and related co-morbidity^17^. In every Cox regression model only incident cases of prescription were analysed excluding cases with previous recording to SPDR. In case of MetS, men who were prescribed to all three medicines as described above, were excluded from analysis. Prevalent cases of TRT prescription were excluded from analysis as were all men treated for opioid addiction (since these treatments affect the function of the hypophysis).

Due to lack of information on the origin of the used gametes before 2007 (before the start of the Q-IVF register) it could not be concluded with highest certainty whether the child was the first to each father and whether previous children were born with donated gametes or not. Therefore, given the average community interval of 2 years between children^18^, a sensitivity analysis omitting the first two years of the study window was done.

## Results

During the period January 1st 2007–31st December 2014, 410,119 men fathered children in Sweden. Among them 390 had used donated spermatozoa and 7698 became fathers by use of ICSI with own spermatozoa. Mean (SD) age of the fathers with donated sperm was 36.6 (5.26) years, 32.0 (6.33) years for the group of fathers who conceived spontaneously and 35.8 (5.4) for ICSI treated men. Mean follow up for the cohort was 4.73 (2.29) years. Table 1 shows the baseline characteristics of the cohort.

**Table 1:** Baseline characteristics of the cohort and subsequent prescription of medicine among the study groups.

	Fatherhood with donated spermatozoa N = 390	Fatherhood with own spermatozoa	
		ICSI N = 7698	Natural conception N = 402,031
**Age: mean (SD)**	36.57 (5.23)	35.8 (5.4)	32.02 (6.33)
**Educational level**			
≤ 10 years	26/6.7%	558/ 7.2%	45,822/11.4%
11–14 years	202/51.8%	3719/48.3%	203,124/50.5%
≥ 15 years	160/41.0%	3372/ 48.3%	14,664/36.4%
Missing data	2/0.5%	49/0.8%	6438/1.6%
**History of cancer before child conception**	36/9.2%	270/3.5%	2338/0.6%
**DM**			
After conception^a^	13/3.3%	178/2.3%	5707/1.4%
Before conception	8/2.05%	89/1.2%	2124/0.5%
**AHT**			
After conception^a^	37/9.5%	642/8.3%	17,249/4.3%
Before conception	22/5.6%	272/3.5%	5125/1.3%
**DLE**			
After conception^a^	30/7.7%	356/4.6%	10,063/2.5%
Before conception	14/3.6%	155/2%	2875/0.7%
**MetS**			
After conception^a^	8/2.05%	67/0.9%	1128/0.3%
Before conception	2/0.5%	19/0.2%	281/0.07%
**TRT**			
After conception^a^	21/309/6.8%	93/7420/1.25%	1167/407,847/0.28%

*DM* (medicines used for the treatment of) diabetes mellitus, *AHT* antihypertensive treatment, *DLE* (medications used for treatment of) dyslipidaemia, *MetS* metabolic syndrome, *TRT* testosterone replacement therapy.

^a^Includes only incident cases.

### Risk of prescription of medicines for MetS among fathers treated with donated spermatozoa

When compared to naturally conceiving fathers, men who fathered a child with donated spermatozoa had higher risk for prescription to drugs for DLE (HR: 2.08; 95%CI: 1.27–3.39; *p* = 0.003). This was not true in comparison to ICSI treated (HR: 1.49; 95%CI: 0.87–2.56; *p* = 0.15). HR was higher for MetS treatment when compared to ICSI treated men (HR: 3.32; 95%CI: 1.41–7.87; *p* = 0.006) but not to the naturally conceiving fathers (HR: 1.44; 95%CI: 0.2–10.29; *p* = 0.77). No evidence for increased risk was shown for DM and AHT prescriptions (DM: HR: 1.32; 95%CI: 0.55–3.17; *p* = 0.54; and HR: 1.78; 95%CI: 0.72–4.41; *p* = 0.26) and (AHT: HR: 1.23; 95%CI: 0.74–2.03; *p* = 0.42 and HR: 1.10; 95%CI: 0.65–1.85; *p* = 0.72) for comparisons with naturally conceiving and ICSI fathers, respectively.

### Risk of prescription of TRT among fathers treated with donated spermatozoa

**Figure 1:**
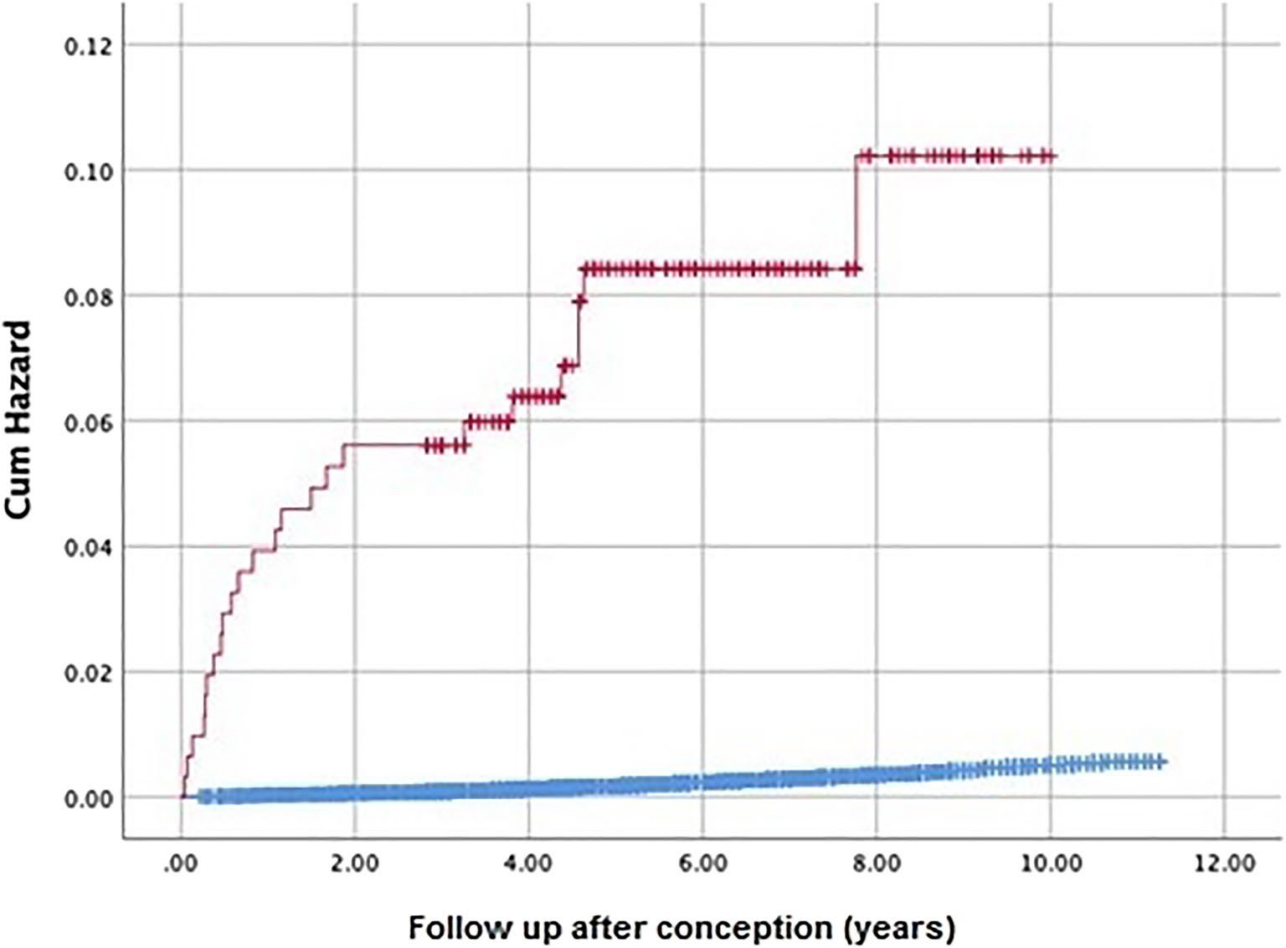
Kaplan Meier curve of testosterone replacement therapy given to fathers treated with donated sperm as compared to spontaneously conceiving fathers. Red line denotes fatherhood with donated spermatozoa, blue line—control group.

Men who fathered a child with donated spermatozoa had higher risk for prescription of TRT when compared to both naturally conceiving and ICSI treated men (HR: 18.14; 95%CI: 11.71–28.10; *p* ≪ 0.001 and HR: 5.10; 95%CI: 3.15–8.27; *p* < 0.001, respectively, Fig. 1).

### Sensitivity analysis omitting the first two years of the follow up

The sensitivity analysis showed similar associations. HRs for TRT, MetS and DLE were higher among men treated with donated sperm when compared to ICSI treated (HR: 7.45; 95%CI: 4.36–12.7; HR 3.26; 95%CI: 1.26–8.41, HR 1.99; 95%CI: 1.00–3.48 accordingly) and TRT and MetS for naturally conceiving (HR 28, 6, 95%CI: 16.5–49.7; HR 4.35; 95%CI: 1.40–13.56; but not for DLE (HR 1.74; 95%CI: 0.90–3.35). Risk estimates of AHT and AD medicines were not statistically significant neither when compared to ICSI treated (HR1.195%CI: 0.62–1.99; HR 2.03; 95%CI: 0.81–5.10) nor when compared to spontaneously conceiving fathers (HR 1.15; 95%CI: 0.65–2.03; HR: 1.49; 95%CI: 0.62–3.6).

## Discussion

Our study has shown that men who became fathers with donated spermatozoa, post-conception, do have a statistically significantly elevated risk for having prescribed DLE and TRT, as compared to those conceiving naturally. Even when compared to ICSI-treated men conceiving with their own spermatozoa, the men who used donated sperm had higher risk for TRT and MetS prescription.

Research on men with NOA or severe olizoospermia and associated health disturbances is scarce. Jensen et.al showed that sperm count and semen quality can serve as health marker suggesting stepwise decrease of life expectancy with worsening of the sperm parameters^4^. Glazer et al. conducted a population-based study in Denmark, showing significantly higher mortality rates in azoospermic men^1^. However, results should be interpreted with caution due to inclusion of 8.7% men with aspermia in the azoospermic group. Men with aspermia have often another underlying medical condition, different to those with to NOA. Nevertheless, the mentioned study is the among the largest to date evaluating the health risks among azoospermic and severely olizoospermic men. A study from US showed higher mortality and morbidity among men with sperm counts lower than 15 × 10^6^/mL^19,20^. However, azoospermic men were not evaluated separately.

By using a population-based registry data similar to the one used in the current study, previous papers evaluated the rate of prescription of medicines for MetS and TRT among men who father children in Sweden with help of intracytoplasmic sperm injection^15,16^. This is the most powerful tool in assisted reproduction developed originally for the treatment of severe cases of male infertility. These men showed higher risks for MetS and TRT but with considerably lower risk estimates than those found for fathers using donated sperms. Thus, it seems that men using donated sperms are at even higher health risk than ICSI fathers who, in general, have severe reduction of semen quality^15^.

Bobjer et al. investigated 65 men with NOA undergoing testicular biopsy. Almost 47% of the subjects presented with hypogonadism which was associated with disturbed lipid profile^7^. These results seem in agreement with our observations.

Possible explanation for our findings might be the inclusion of men who have Klinefelter syndrome—the most frequent genetical cause of NOA^6^. This group of men is known to have higher cardiovascular and cardiometabolic morbidity and mortality according to most studies^21–23^ despite some showing lower mortality rates for e.g. ischaemic heart disease^24^. However, it is unlikely that our results can be explained entirely by inclusion of men with Klinefelter syndrome. These men account for only 15% of the men with azoospermia^6^ and are often on TRT since early adulthood. In our study, only those not prescribed relevant medications prior to the conception were included.

Association between severe male infertility, cryptorchidism, testicular cancer has been suggested to represent testicular dysgenesis syndrome (TDS)^25^. According to this hypothesis, TDS is consequence of interplay between adverse genetic and environmental exposures during the in-utero development of the male embryo. It makes it therefore plausible to assume that TDS might lead to both infertility and various adult-onset diseases this effect being mediated by hypogonadism or other not yet identified mechanism.

Shortcoming of the presented study is the lack of information on the clinical indication for use of sperm donation. Some men with non-obstructive azoospermia and cases with risk of transmission of paternal genetic disease to the child might be included in the group of men who became fathers by sperm donation. Use of donated sperm to conceive as proxy for men with NOA or severe oligozoospermia implies risk of some misclassification of study subjects. In the manuscript, those two subgroups have been categorized as one entity because the latter condition is often associated with high rates of morphologically altered sperm and poor fertilization. It has been shown that very low sperm counts have a negative impact on ICSI outcomes^26^. In the period 2007–2014, the access to micro-TESE in Sweden was very limited. Thus, there is reason to believe that men in couples who conceived using donated spermatozoa are not exclusively azoospermic but a proportion of them might be severely oligozoospermic. In the clinical practice it is common that some of these couples, after numerous failed attempts, decide to continue the fertility treatment with donated spermatozoa.

In many countries there is an unregulated internet market for donor spermatozoa for couples who wish more privacy than what can be offered by the public health service. However, in Sweden usage of donated gametes is regulated by law and men and women have to undergo a meticulous psychosocial examination prior to further treatment. During the time period 2007–2014 only public clinics were allowed to perform treatments with donated gametes. When it comes to the unregulated market, however, no register data are available. Furthermore, some of the couples with azoospermic or severely oligozoospermic male partner might have decided not to have children or proceed with adoption. Those two groups will not be represented in our study, since we only included fathers to children born in Sweden 2007–2014. On the other hand, a proportion of azoospermic or severely oligozoospermic men were included in the control group if they conceived using testicular spermatozoa.

It can be even speculated that some cases of obstructive azoospermia, including failed vasectomy reversal, might have undergone treatment with donated spermatozoa. However, in the majority of cases those couples succeed to achieve pregnancy using gametes obtained by extraction from epididymis or testes. Finally, androgen abusers using illicit non-prescribed androgens causing azoospermia may not be excluded by the exclusion for prescribed testosterone usage.

Thus, although there is reason to believe that although our set up implies some misclassification, all of the caveats would lead to underestimation of HRs rather than to statistically significant findings reported by us. Another limitation is the lack of possibility to differentiate if it is the disease (MetS) and its possible risk factors that causes azoospermia or visa versa. In this relation it would have been relevant to have information on social factors as a proxy for e.g. life style patterns. This information is not available but previous research has shown that social factors have little effect on the association between low semen quality and health^27^. Furthermore, there are no data showing that lifestyle factors such as smoking, physical activity, alcohol and eating habits are disproportionately distributed between fathers who use donated or own spermatozoa to sire offspring.

However, it should be kept in mind that our HR estimates may be an underestimation of the real risk of testosterone deficiency and/or metabolic disturbances. Both conditions may give rather mild and uncharacteristic symptom and can, therefore, easily be overlooked. A recent report showed, that obese infertile men with normal metabolic parameters have higher risk for primary and secondary hypogonadism when compared to metabolically healthy normal weighted infertile men^28^. This illustrates the importance of implementing routine measurement of hormonal and metabolic parameters in infertile men—crucial for prevention of serious, long-term consequences.

## Conclusion

The presented analysis shows that fathers to children conceived by use of donated spermatozoa are at significantly increased risk for testosterone deficiency and cardio-metabolic disturbances. Our finding stresses the need of stringent follow up and inclusion of these men in health prevention programs. Our results necessitate further studies on health implications of NOA and severe olizoospermia beyond reproductive failure.

## Data Availability

The data that support the findings of this study are available from Swedish National Board of Health and Welfare, but restrictions apply to the availability of these data, which were used under license for the current study, and so are not publicly available. Data are however available from the authors upon reasonable request and with permission of Swedish National Board of Health and Welfare.
